# Functional Dissection of the Nascent Polypeptide-Associated Complex in *Saccharomyces cerevisiae*

**DOI:** 10.1371/journal.pone.0143457

**Published:** 2015-11-30

**Authors:** Ann-Kathrin Ott, Lisa Locher, Miriam Koch, Elke Deuerling

**Affiliations:** 1 Molecular Microbiology, Department of Biology, University of Konstanz, 78457, Konstanz, Germany; 2 Konstanz Research School of Chemical Biology, University of Konstanz, 78457, Konstanz, Germany; University of Pittsburgh, UNITED STATES

## Abstract

Both the yeast **n**ascent polypeptide-**a**ssociated **c**omplex (NAC) and the Hsp40/70-based chaperone system RAC-Ssb are systems tethered to the ribosome to assist cotranslational processes such as folding of nascent polypeptides. While loss of NAC does not cause phenotypic changes in yeast, the simultaneous deletion of genes coding for NAC and the chaperone Ssb (*nacΔssbΔ*) leads to strongly aggravated defects compared to cells lacking only Ssb, including impaired growth on plates containing L-canavanine or hygromycin B, aggregation of newly synthesized proteins and a reduced translational activity due to ribosome biogenesis defects. In this study, we dissected the functional properties of the individual NAC-subunits (α-NAC, β-NAC and β’-NAC) and of different NAC heterodimers found in yeast (αβ-NAC and αβ’-NAC) by analyzing their capability to complement the pleiotropic phenotype of *nacΔssbΔ* cells. We show that the abundant heterodimer αβ-NAC but not its paralogue αβ’-NAC is able to suppress all phenotypic defects of *nacΔssbΔ* cells including global protein aggregation as well as translation and growth deficiencies. This suggests that αβ-NAC and αβ’-NAC are functionally distinct from each other. The function of αβ-NAC strictly depends on its ribosome association and on its high level of expression. Expression of individual β-NAC, β’-NAC or α-NAC subunits as well as αβ’-NAC ameliorated protein aggregation in *nacΔssbΔ* cells to different extents while only β-NAC was able to restore growth defects suggesting chaperoning activities for β-NAC sufficient to decrease the sensitivity of *nacΔssbΔ* cells against L-canavanine or hygromycin B. Interestingly, deletion of the **ub**iquitin-**a**ssociated (UBA)-domain of the α-NAC subunit strongly enhanced the aggregation preventing activity of αβ-NAC pointing to a negative regulatory role of this domain for the NAC chaperone activity *in vivo*.

## Introduction

**Fig 1:**
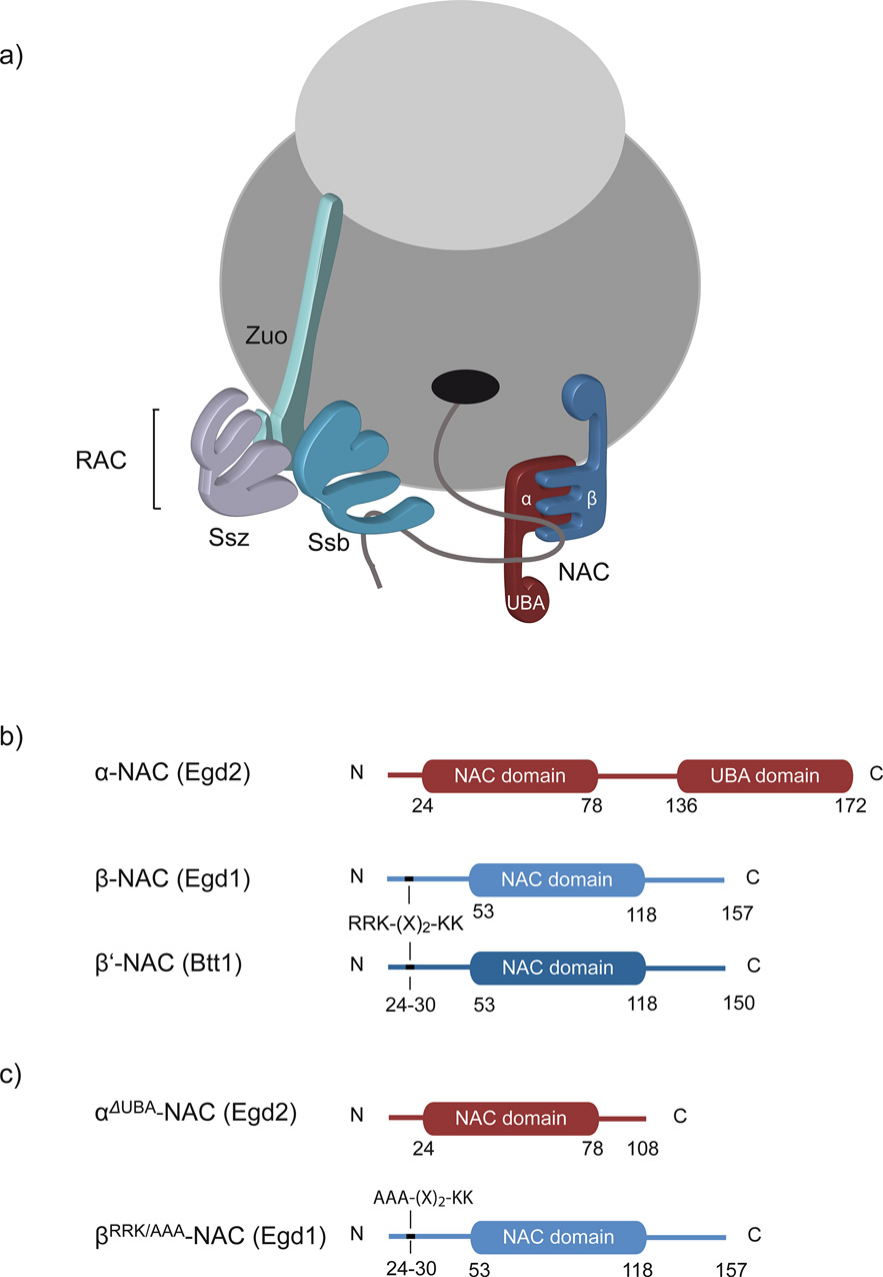
Ribosome-associated chaperones from *S*. *cerevisiae*. a) The Hsp70/Hsp40-chaperone system that consists of RAC (Ssz and Zuo), shown in purple and light green, and Ssb, shown in light blue, forms a functional triad at the ribosome. In addition, β-NAC (shown in blue) and α-NAC (shown in red) that contains a C-terminal UBA (ubiquitin-associated) domain constitute the stable heterodimeric αβ-NAC complex which binds to the ribosome via the ribosome-binding motif in the β-subunit. Both, NAC and Ssb can interact directly with the nascent chain. b) Schematic representation of the different NAC subunits. α-NAC (shown in red) contains a NAC domain and a UBA domain. Besides the NAC domain the two different β-subunits (shown in light and dark blue) also contain a conserved ribosome-binding motif present in their N-termini. c) Schematic drawing of the two NAC mutants investigated in this study. α^ΔUBA^-NAC (shown in red) lacks the C-terminal UBA domain and part of the linker region. In β^RRK/AAA^-NAC (shown in blue) the conserved RRK-(X)_2_-KK motif was mutated to AAA-(X)_2_-KK to abolish ribosome binding.

The folding of newly synthesized proteins requires the assistance of molecular chaperones. At the forefront are ribosome-associated chaperones, which contact nascent polypeptides to control early protein folding processes and to prevent aggregation or degradation of newly synthesized proteins [1, 2]. Yeast ribosomes are transiently associated with two different types of chaperone systems. One is a Hsp70/Hsp40-based chaperone system consisting of the **r**ibosome-**a**ssociated **c**omplex (RAC), a heterodimer formed by Zuo(tin) and Ssz, and Ssb. The second system is the **n**ascent polypeptide-**a**ssociated **c**omplex (NAC). Both systems bind transiently to the large ribosomal subunit for interaction with nascent polypeptides early during protein biogenesis (Fig 1A).

NAC reversibly binds to ribosomes and is described as the first factor that interacts with nascent polypeptides emerging from the ribosome to prevent them from incorrect interactions [3]. The complex is widely conserved from archaea to man, however, archaea only have a homodimeric NAC formed by two α-NAC subunits while yeast and higher eukaryotes mainly form a stable αβ-NAC heterodimer (Fig 1A). Crystal structures of NAC deletion variants from archaea and humans suggest that the two NAC subunits dimerize via their homologous six-stranded β-barrel-like NAC domains (Fig 1B). Only α-NAC contains an additional UBA (ubiquitin-associated) domain with unknown function at its C-terminus (Fig 1B) [4–6]. Ribosome binding of eukaryotic NAC is mediated by the N-terminus of β-NAC involving a conserved ribosome-binding motif (Fig 1B) and probably an adjacent helix element [7–9]. Mutations in this region of β-NAC (β^RRK/AAA^-NAC, Fig 1C) diminish ribosome binding of the entire complex [9]. Crosslinking data suggest different binding sites for NAC on the ribosome including the ribosomal proteins Rpl31 (eL31) and Rpl25 (uL23), which have been shown to be functional docking sites for other ribosome-attached factors as well, including Zuotin or bacterial Trigger Factor, SRP, and the ER-translocon, respectively [5, 9–11]. Moreover, both subunits of αβ-NAC crosslink to nascent polypeptides [7], suggesting that both can contact substrates, but the substrate binding sites in the individual subunits have not been elucidated yet.

In contrast to other eukaryotic organisms, the *Saccharomyces cerevisiae* genome encodes three NAC subunits: One α-NAC subunit encoded by the *EGD2* gene and two alternative β-NAC paralogues, β-NAC and β’-NAC, encoded by the *EGD1* and the *BTT1* gene, respectively. Hence, two different types of heterodimers, αβ-NAC and αβ’-NAC, are formed in yeast with potentially different substrate pools and functions [12]. However, β’-NAC *(BTT1)* is approximately 100-fold less expressed than β-NAC and thus, the heterodimeric αβ-NAC seems to be the dominant species in yeast. Moreover, it is suggested that to a minor extent also homodimers of both subunits exist *in vivo* [10, 13, 14].

While yeast NAC is not important for growth, NAC is essential in higher eukaryotes and a loss of its function induces early embryonically lethal phenotypes or developmental defects in mice, fruit flies, and *C*. *elegans* [15–17]. Recent data show that NAC is a member of the chaperone network in yeast and in *C*. *elegans* [12, 18, 19]. The simultaneous deletion of all three NAC genes in yeast does not cause growth defects while the combined deletion of NAC genes together with genes encoding the Ssb chaperone (*SSB1* and *SSB2*) leads to synthetic defects including impaired growth under protein folding stress, enhanced aggregation of newly synthesized proteins and accelerated defects in ribosomal biogenesis and translation [18]. Two recent studies revealed the functions of NAC in metazoans [19, 20]. Depletion of NAC in *C*. *elegans* results in enhanced protein aggregation of folding-sensitive polyQ proteins. Moreover, NAC was shown to be associated with heat-induced aggregates of firefly luciferase in transgenic *C*. *elegans* strains and loss of NAC prevents the efficient re-solubilization of aggregated luciferase at permissive temperature in these animals [19]. In addition, NAC plays an essential role as a negative regulator in cotranslational protein targeting to the ER in metazoans. Bound to ribosomes, NAC shields the high affinity binding site of ribosomes for the Sec61 translocon and thereby prevents incorrect ribosome-nascent chain complexes from association with the ER-translocon and the erroneous import of incorrect cargo in the ER [20]. However, this function is less well understood in yeast where *in vivo* studies showed no aberrant translocation phenotype upon NAC deletion [10, 14, 21], perhaps due to the fact that yeast cells use a distinct posttranslational ER targeting system in addition.

In this study we set out to better understand the function of NAC and its individual subunits in yeast. To this end, we expressed individual subunits or heterodimeric complexes of NAC in cells lacking NAC and Ssb and investigated which subunits of NAC are potent to complement the phenotypic changes of *nacΔssbΔ* cells.

## Results

### The β-subunit of NAC is essential and sufficient to ameliorate growth

**Fig 2:**
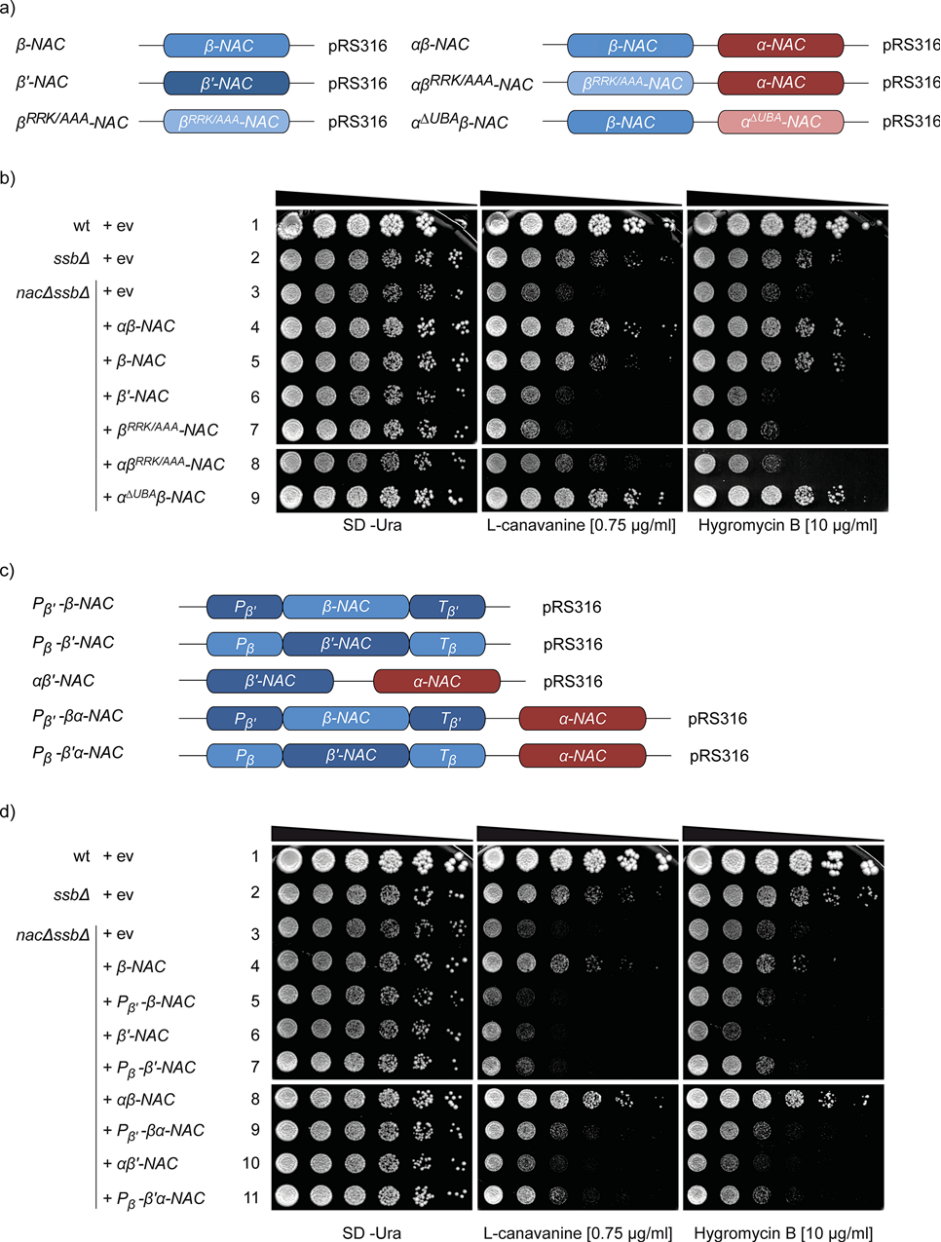
The αβ-NAC complex and β-NAC under control of their endogenous promoter complement the growth defect of *nacΔssbΔ* cells. a) Schematic drawing of the different plasmid-encoded NAC constructs used in this study. Plasmids encoding wild type (wt) and mutant αβ-NAC, either alone or in complex, were cloned in the vector backbone pRS316 reported by [18]. b) Growth analysis of wt and mutant yeast cells expressing different NAC versions from plasmids as indicated. Serial dilutions were spotted on synthetic complete media without uracil (SD-Ura) containing the indicated drugs. When cells were plated on the arginine analogue L-canavanine, arginine was omitted. The cells were incubated for 3 days at 30°C. c) The promoter (P)—and terminator (T)- regions of *EGD1* were replaced with the corresponding regions of *BTT1* and *vice versa* and cloned in the vector backbone of pRS316 with or without *EGD2*. *BTT1* under its endogenous promoter and terminator was also cloned into pRS316 together with *EGD2*. d) Growth analyses were performed as described in b).

The loss of NAC does not result in a growth phenotype, while *nacΔssbΔ* cells lacking all NAC and Ssb chaperone encoding genes (*EGD1Δ*, *EGD2Δ*, *BTT1Δ*, *SSB1Δ*, *SSB2Δ)* show a strong growth deficiency compared to wild type (wt) or *ssbΔ* cells at 30°C, in particular in the presence of drugs which impair protein synthesis or folding like the arginine analogue L-canavanine or the translation inhibitor hygromycin B [18]. To understand which NAC subunits are essential for growth, we expressed NAC subunits encoded on centromeric plasmids (Fig 2A) individually or in combinations and tested their ability to complement the growth defects of *nacΔssbΔ* cells (Fig 2B). Expression of NAC genes was driven by the respective authentic promoter (Fig 2A) and protein levels were probed by Western blotting revealing similar expression levels as in the wt (S2B Fig).

Expression of the αβ-NAC heterodimer complemented the growth defect of *nacΔssbΔ* cells in the presence of L-canavanine or hygromycin B (Fig 2B, lane 4). This finding is in agreement with an earlier report [18]. Interestingly, also the expression of β-NAC alone as well as in combination with an α-NAC variant lacking the C-terminal 64 amino acid residues including the UBA domain (called hereafter α^ΔUBA^-NAC, see Fig 1C) ameliorated growth of *nacΔssbΔ* cells in the presence of both drugs (Fig 2B, lanes 4, 5 and 9). In contrast, the expression of the ribosome-binding mutant β^RRK/AAA^-NAC alone (Fig 1C) or in combination with α-NAC did not decrease sensitivity to L-canavanine or hygromycin B compared to the vector control (Fig 2B, lanes 3, 7 and 8). This data suggests that expression of the β-NAC subunit is sufficient to restore growth defects of *nacΔssbΔ* cells back to the growth properties of *ssbΔ* cells. Based on the finding that αβ^RRK/AAA^-NAC did not restore growth, we conclude that the α-NAC subunit is not sufficient to promote growth and ribosome-binding of β-NAC is crucial for the activity of αβ-NAC. In contrast to β-NAC, the expression of β’-NAC did not ameliorate growth of *nacΔssbΔ* cells in presence of L-canavanine or hygromycin B (Fig 2B, lane 6) implying that the paralogous β’-NAC subunit is either functionally distinct from β-NAC in yeast or, due to its low expression, the level of β’-NAC is not sufficient to support growth under these conditions.

### Expression levels of β-NAC but not β’-NAC are important for growth

The expression ratio of β-NAC *(EGD1)* and β’-NAC *(BTT1)* in yeast cells is about 100:1 [13, 14]. To investigate whether the expression levels of β-NAC and β’-NAC are important for their ability to complement the growth defects of *nacΔssbΔ* cells, we exchanged the promoter and terminator region of *BTT1* with regions from the *EGD1* gene and *vice versa* (Fig 2C) and tested these constructs in *nacΔssbΔ* cells (Fig 2D).

When the *BTT1* promoter and terminator regions were fused to the *EGD1* gene, which results in strongly reduced *β-NAC* mRNA levels (S1A Fig), the growth defect of *nacΔssbΔ* cells on plates containing translation inhibitory drugs could not be complemented (Fig 2D, compare lane 4 with lane 5). Similarly, low expression of β-NAC in combination with wt levels of α-NAC did no longer enhance growth under these conditions (Fig 2D, lane 9). We conclude from these results that the high expression level of β-NAC is crucial for enhanced growth of *nacΔssbΔ* cells on plates containing L-canavanine or hygromycin B.

In turn, expression of β’-NAC under control of the *EGD1* promoter and terminator led to significantly higher levels of *BTT1* mRNA (S1B Fig). The high level expression of β’-NAC alone or in combination with wt levels of α-NAC enhanced growth of *nacΔssbΔ* cells on plates containing drugs compared to αβ’-NAC expressing cells (Fig 2D, compare lanes 6 and 7, 10 and 11), however, only slightly compared to cells expressing β-NAC or αβ-NAC (Fig 2D, lanes 4 and 8). It should be mentioned that the expression levels of the β-NAC variants with exchanged promoter and terminator regions were tested on the mRNA level due to the lack of specific antibodies. Thus, we cannot exclude that some variations on the protein level may contribute to the observed effects as well.

In summary, the data suggest that the paralogous ribosome-associated β-NAC and β’-NAC execute distinct functions *in vivo* even when expressed at similar levels.

### Ribosomal defects in *nacΔssbΔ* cells are suppressed by αβ-NAC

**Fig 3:**
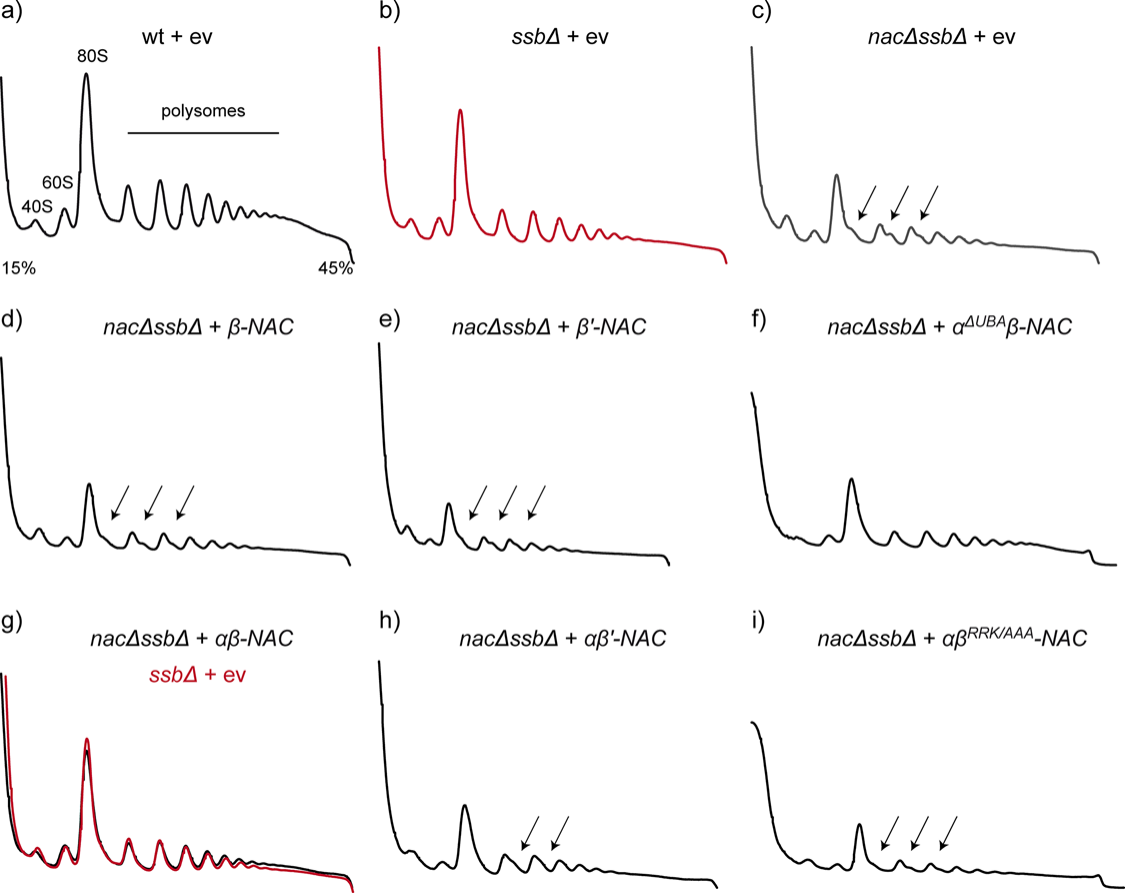
Halfmer formation of *nacΔssbΔ* knockout cells can be prevented by expression of *αβ-NAC*. a-i) Polysome profiles derived from wild type (wt) or mutant yeast cells. Absorbance traces at 254 nm are shown. Cells were grown to an optical density (OD_600_) of 0.8 in SD-Ura medium. 10 A_260_ units of lysates of indicated cells were loaded onto 15–45% linear sucrose gradients to isolate ribosomal fractions (40S, 60S, 80S and polysomes) as indicated by centrifugation and subsequent fractionation. Polysome profiles show: a) wt + empty vector (ev), b) *ssbΔ* cells + ev, c-i) *nacΔssbΔ* cells + ev (c), + *β-NAC* (d), *β’-NAC* (e), *α* ^*ΔUBA*^ *β-NAC* (f), *αβ-NAC* (g) + *αβ’-NAC* (h) and *αβ* ^*RRK/AAA*^ *-NAC* (i) The profiles are representative for three independent experiments.

Previous studies revealed that *nacΔssbΔ* cells show a defect in ribosome biogenesis leading to the formation of ribosomal halfmers and a reduced translational activity [18]. This defect in ribosome biogenesis can be investigated by separating total cell lysate on a sucrose gradient using ultracentrifugation and subsequent fractionation of the gradient monitoring ribosomal species by measuring the absorption at 254 nm. The peak heights of the absorption traces detected at 254 nm could be used as sensitive indicators for the levels of each ribosomal species because equal absorption units of the samples were loaded. Thereby, the shoulder in the 80S and polysome peaks of the double knockout cells represents the presence of ribosomal halfmers in such fractionation experiments (Fig 3C, arrows). Such halfmers consist of an uncomplexed 40S subunit bound to the mRNA and are typically caused by an impaired balance of 40S and 60S ribosomal subunits due to defects in the assembly of 60S particles [22, 23]. Indeed, higher levels of 40S subunits were detected in *nacΔssbΔ* cells compared to the wild type. Moreover, the 80S monosome and polysome peaks were significantly reduced and ribosomal halfmers were present in *nacΔssbΔ* cells compared to wt cells indicating the reduced translational activity (Fig 3A and 3C). As reported earlier [18], these ribosomal defects are clearly less pronounced in cells lacking only Ssb (Fig 3B).

**Fig 4:**
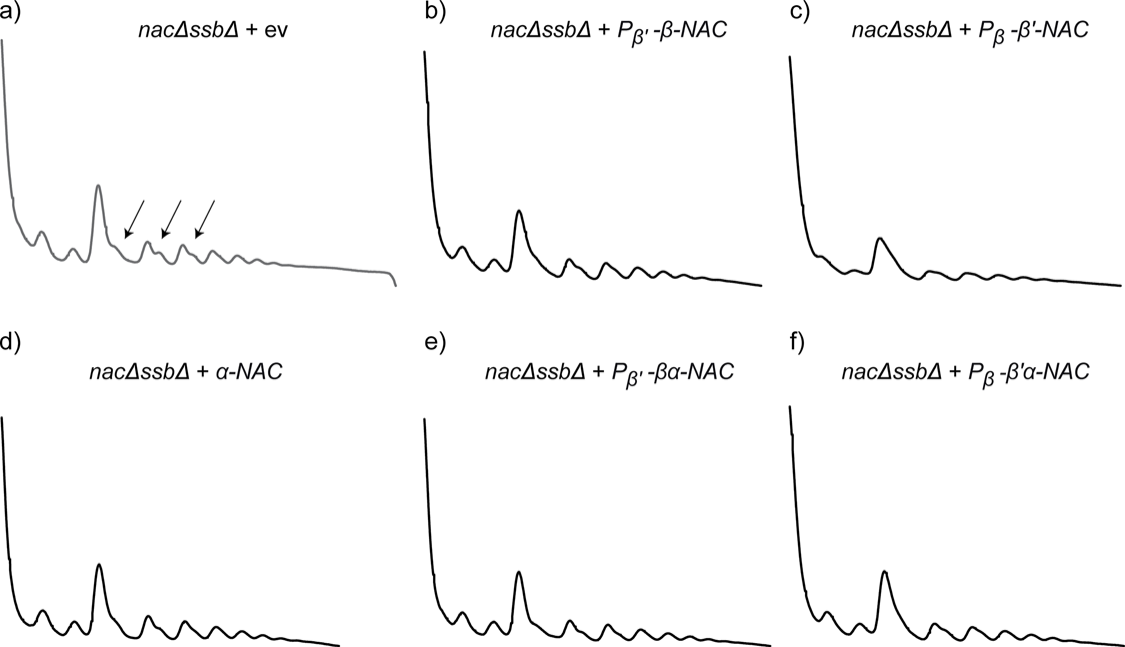
Expression levels of *β-NAC* are important for complementation of *nacΔssbΔ* halfmers. Polysome profiling with wild type (wt) or mutant yeast cells. Absorbance traces at 254 nm are shown. 10 A_260_ units of lysates of indicated yeast cells were loaded onto 15–45% linear sucrose gradients similar to Fig 3. a) Polysome profile of *nacΔssbΔ* cells + empty vector control (ev) (in grey). b) and c) Complementation of *nacΔssbΔ* cells with promoter-swapped *β-NAC* constructs alone. d) *nacΔssbΔ* cells expressing α-NAC alone. e) and f) Polysome profiles of *nacΔssbΔ* cells expressing promoter-swapped β-NAC constructs in combination with α-NAC. Arrows indicate halfmers. The profiles are representative for three independent runs.

To test which subunit(s) of NAC complement(s) the ribosomal defects, ribosome profiles were generated from *nacΔssbΔ* cells expressing different NAC variants. We found that in contrast to the growth analysis, only the expression of the αβ-NAC heterodimer could restore the ribosome biogenesis defects observed in *nacΔssbΔ* cells (Fig 3G). This is demonstrated by a decreased amount of halfmers, a reduced 40S peak and enhanced 80S and polysome peaks resulting in a profile that is similar to *ssbΔ* cells (Fig 3B, 3C and 3G). Importantly, expression of the ribosome-binding deficient αβ^RRK/AAA^-NAC version did not suppress these deficiencies in ribosome biogenesis and translation (Fig 3I). Moreover, neither expression of β-NAC nor β’-NAC alone cured the ribosomal defects (Fig 3D and 3E). A small reduction in the amount of halfmers could be observed upon expression of αβ’-NAC (Fig 3H). We also investigated whether the expression levels of β-NAC and β’-NAC are crucial for the suppression of ribosomal defects in *nacΔssbΔ* cells (Fig 4). High level expression of β’-NAC driven by the *EGD1* promoter and terminator elements with or without coexpression of α-NAC resulted also in a very mild reduction of ribosomal halfmers confirming again that the β’-NAC subunit and consequently also the αβ’-NAC heterodimer are functionally distinct from β-NAC and αβ-NAC, respectively, even when expressed at similar levels (Fig 4C and 4F). Moreover, a reduced expression of αβ-NAC did not complement the aberrant translation phenotype (Fig 4B and 4E) and also α-NAC itself could not prevent halfmer formation of *nacΔssbΔ* knockout cells (Fig 4D). Interestingly, the expression of the α^ΔUBA^β-NAC mutant version in *nacΔssbΔ* cells (Fig 3F) reduced the halfmer formation whereas the 80S and polysome peaks were still reduced compared to *ssbΔ* cells, suggesting that the UBA domain functionally contributes to the function of αβ-NAC in translation.

Taken together, these data show that, in contrast to the growth analyses where ribosome-bound β-NAC is sufficient for complementation, exclusively the αβ-NAC heterodimer expressed at high levels is able to support proper ribosome biogenesis and translation in yeast cells.

### NAC is transcriptionally not coregulated with components of the translation apparatus

**Fig 5:**
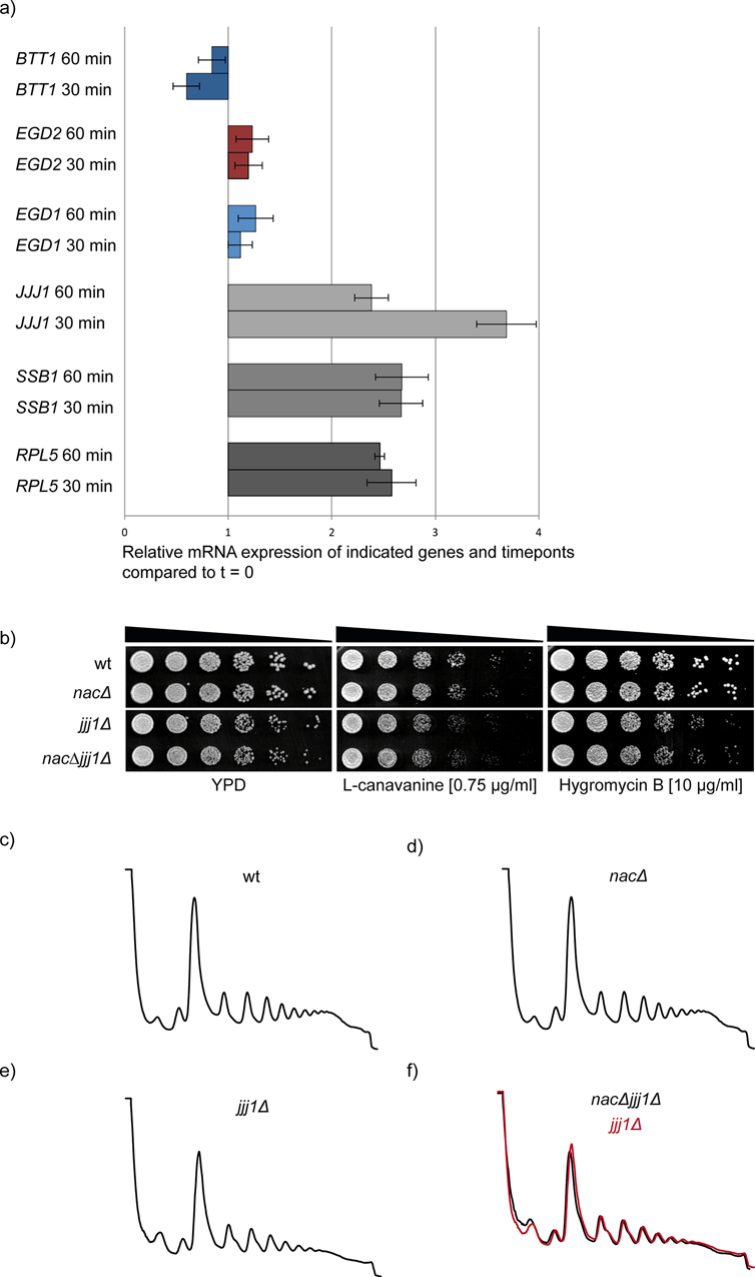
*NAC* is not coregulated with genes encoding ribosomal proteins. a) X-axis: Relative mRNA levels of indicated genes and time points compared to timepoint zero (t = 0, before glucose addition) and normalized to an internal control (housekeeping gene). Cells were harvested at 0 min, 30 min and 60 min after glucose addition and mRNA was extracted. cDNA was obtained by reverse transcription and used for qRT-PCR. b) Serial dilutions of wild type (wt) and chaperone mutant cells were spotted on YPD plates and plates containing the indicated drugs for growth analysis. When cells were plated on the arginine analogue L-canavanine, arginine was omitted. The cells were incubated for 3 days at 30°C. c) Polysome profiles of wt and mutant cells. 10 A_260_ units of lysates of indicated yeast strains were loaded onto 15–45% linear sucrose gradients as shown in Fig 3. The profiles are representative for three independent runs.

The expression of genes encoding proteins involved in ribosome biogenesis is often coregulated with genes coding for ribosomal proteins [24]. This has been reported also for the ribosome-associated chaperone Ssb [25]. As the ribosomal biogenesis and translation defects of cells lacking NAC and Ssb are more pronounced than in the cells lacking only Ssb, we wondered whether the genes *EGD1*, *BTT1* and *EGD2* coding for NAC are also coregulated with ribosomal genes. In a previous study from Albanèse et al. [26] where transcriptional analysis of gene expression in response to environmental stress, e.g. heat shock or nitrogene depletion, was performed, NAC was found to be corepressed together with components of the translational apparatus and ribosome biogenesis chaperones such as Ssb and RAC. To further address this question under non-stress conditions, wt cells were grown in medium containing glycerol as carbon source until they reached an OD_600_ of 0.6 (time point zero). Then the cells were washed and transferred into medium containing glucose because ribosomal genes are upregulated upon carbon upshift from glycerol- to glucose-containing medium. Total RNA was isolated after various time points and followed by quantitative real-time PCR. We found, in agreement with earlier studies [25], that the mRNAs of *SSB1* and the ribosomal protein *RPL5* as well as the mRNA of the ribosome biogenesis factor *JJJ1* were upregulated about 2- to 3.5-fold upon carbon shift (Fig 5A). However, no significantly enhanced transcription of mRNA coding for any of the three NAC subunits was detected. The mRNA levels of *EGD1* and *EGD2* remained almost constant in comparison to *SSB1* or *JJJ1* and the mRNA level of *BTT1* was even slightly reduced upon carbon shift (Fig 5A). Hence, NAC is not coregulated with ribosomal proteins under these conditions, which is a typical characteristic for ribosomal biogenesis factors and chaperones directly involved in this process, such as Jjj1 or Ssb. It is known that loss of Jjj1 causes a slow growth phenotype and the combined deletion of the *SSB1*,*2* genes and *JJJ1* results in synthetic lethality [18]. To further investigate the role of NAC in ribosome biogenesis, we generated *jjj1Δ* and *nacΔjjj1Δ* knockout strains to test for a genetic interaction. The *nacΔjjj1Δ* cells lacking Jjj1 and all three genes encoding NAC showed no synthetic growth phenotype compared to *jjj1Δ cells* under the conditions tested (Fig 5B). Ribosome profiles of *jjj1Δ* and *nacΔjjj1Δ* cells (Fig 5C–5F) revealed that the deletion of *JJJ1* resulted in a decrease of 60S subunits and in the appearance of halfmers (Fig 5E), indicating that this strain has a ribosome biogenesis defect as described previously [27, 28]. Loss of NAC in *jjj1Δ* cells did neither enhance the halfmer formation nor cause a further reduction of 60S, 80S or polysome peaks. This suggests that NAC and Jjj1 do not display overlapping functions in ribosome biogenesis and indicates together with the lack of transcriptional coregulation that NAC supports the activity of the translation apparatus by a mechanism distinct from classical ribosome biogenesis factors.

### Suppression of protein aggregation by NAC

**Fig 6:**
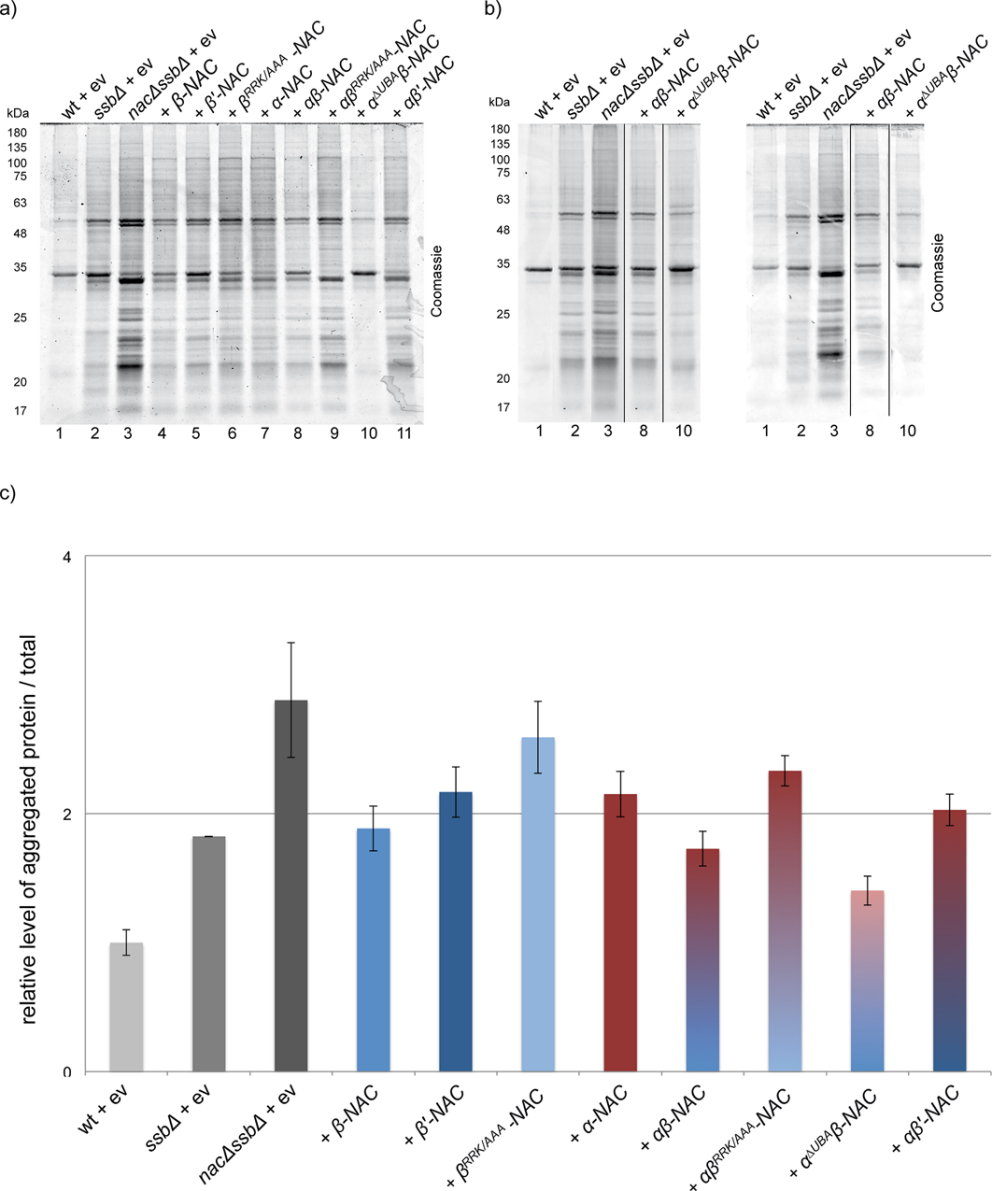
Analysis of protein aggregation in *nacΔssbΔ* suppressed by *NAC* variants. a) 50 OD_600_ units of transformed yeast cells expressing the indicated NAC variants in the logarithmic phase were lysed and the aggregated protein material was isolated by sedimentation. Isolated aggregated fractions were separated by SDS-PAGE and visualized by Coomassie staining. b) Biological replicates of the experiment shown in a) for aggregated proteins of wt, *ssbΔ* and *nacΔssbΔ* cells (lanes 1–3), *nacΔssbΔ* + *αβ-NAC* (lane 8) and + *α* ^*ΔUBA*^ *β-NAC* (lane 10). The experiment was performed as in a). For better visualization the corresponding lanes were cut out from the same SDS-PAGE after Coomassie staining as indicated by black lines. c) Quantification of aggregated material using ImageJ shows the relative level of aggregated protein in relation to total protein amount, normalized to the mean value of wt replicates. Mean ± SD is shown from three experiments (n = 3).

The loss of NAC does not provoke protein aggregation while cells lacking the Ssb chaperone activity accumulate misfolded and insoluble proteins. However, defects in protein folding are much more pronounced in *ssbΔ* cells upon additional loss of NAC suggesting that these two ribosome-associated systems act in overlapping pathways to support the folding of newly synthesized proteins [18]. Therefore, we finally examined if NAC subunits expressed alone or in combination reduce the level of protein aggregation in *nacΔssbΔ* cells. Mutant cells were grown to exponential phase, harvested and after lysis the insoluble protein material was isolated by centrifugation (Fig 6). Equalized total lysates are shown in S2A Fig and served as a loading control. Three biologically independent experiments were conducted for each NAC variant to test the chaperone activity by analyzing the suppression of protein aggregation in *nacΔssbΔ* cells. The data were analyzed using one-way-between-groups ANOVA with post-hoc Tukey test [29] to assess significance. As shown previously, cells lacking Ssb and NAC revealed enhanced levels of insoluble protein material compared to *ssbΔ* cells (Fig 6A, lanes 1–3). We found that expression of αβ-NAC at wt levels significantly reduced protein aggregation in *nacΔssbΔ* cells (Fig 6A, lane 8; quantification in Fig 6C and S1 Table (p = 0)) suggesting that this heterodimer is a potent chaperone *in vivo*. The expression of the single NAC-subunits or of αβ’-NAC ameliorated protein aggregation as well, with some variances compared to the expression of αβ-NAC (Fig 6A, lanes 4, 7, 11 and S1 Table). This suggests that chaperone activity can be displayed by individual subunits and does not necessarily rely on the heterodimeric NAC complex. The chaperone activity of αβ-NAC and β-NAC critically depends on ribosome association as protein aggregation could not be prevented in *nacΔssbΔ* cells expressing the αβ^RRK/AAA^-NAC or β^RRK/AAA^-NAC subunit (Fig 6A, lanes 6 and 9, Fig 6C, S1 Table (p = 0.24 and 0.921, respectively)).

Surprisingly, the most potent NAC version in preventing protein aggregation was α^ΔUBA^β-NAC (Fig 6A, lane 10 and S1 Table, p = 0, highly significant). Fig 6A and 6B show the SDS-PAGES of the isolated and Coomassie-stained aggregated protein species from *nacΔssbΔ* cells expressing either α^ΔUBA^β-NAC or αβ-NAC in comparison to controls for all three biological replicates. Expression of α^ΔUBA^β-NAC ameliorated protein aggregation even more than αβ-NAC (Fig 6A and 6B; compare lanes 10 and 8) to a level similar to wt cells (S1 Table, p = 0.633, not significant). This data suggests an enhanced chaperone activity for α^∆UBA^β-NAC and suggests that the UBA domain negatively regulates the chaperone activity of αβ-NAC *in vivo*.

## Discussion

The eukaryotic conserved heterodimer αβ-NAC was discovered in 1994 by Wiedmann and colleagues [7] and has since then been studied *in vitro* and *in vivo* to better understand the diverse roles of this complex e.g. in protein folding and transport [2, 30]. NAC is not essential in yeast but displays a strong genetic and functional interaction with the Ssb chaperone as is evident by the amplification of the pleiotropic defects found in cells lacking Ssb upon additional deletion of NAC encoding genes [18]. In contrast to other eukaryotes, *Saccharomyces cerevisiae* encodes besides the universally conserved α-NAC and β-NAC subunits that form the stable and abundant αβ-NAC heterodimer, a second paralogous β’-NAC subunit that forms the alternative αβ’-NAC dimer. In this study, we dissected the functions of the individual NAC subunits and the two different heterodimers by their ability to complement the pleiotropic phenotype of *nacΔssbΔ* cells. In addition, our results demonstrate the importance of ribosome binding of NAC and identify for the first time a potential role for the UBA-domain of α-NAC in regulating the chaperone activity of αβ-NAC.

**Fig 7:**
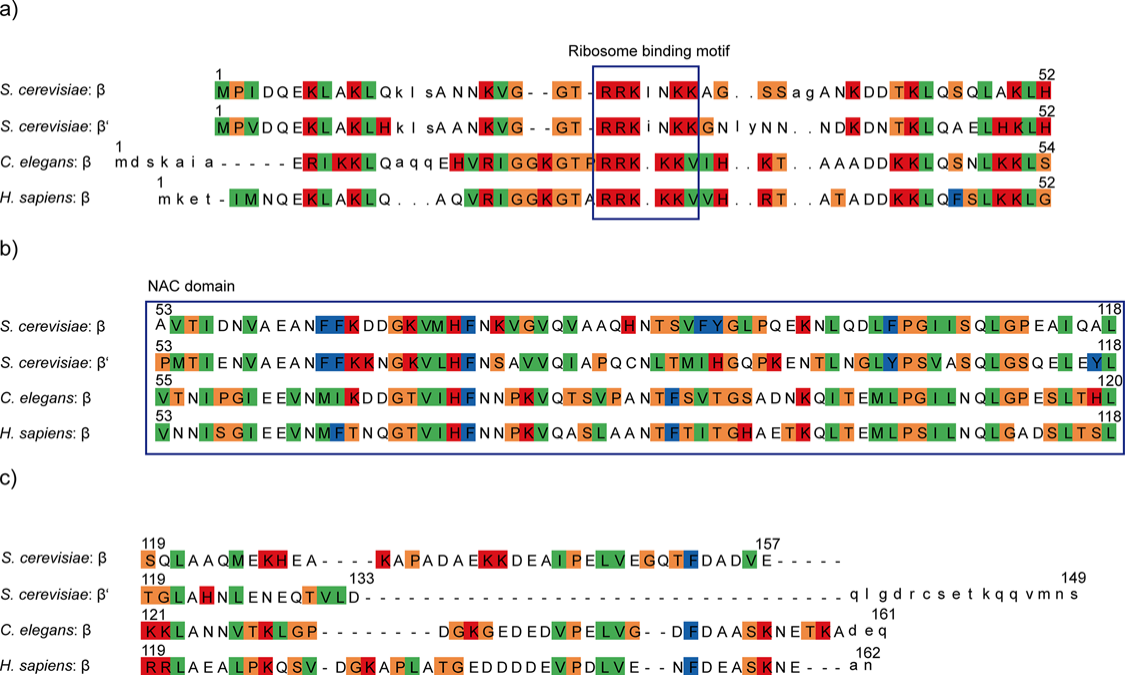
β-NAC and β’-NAC show differences in their C-termini. First, a PSI-BLAST search of the NCBI database was performed. Then the sequences were sorted using CLANS [34] and aligned with the alignment programme muscle [35]. An HMM (http://hmmer.org/) was constructed of the fungi sequences and all sequences were aligned against the HMM. The sequences are shown for a) the N-terminus, b) the NAC-domain and c) the C-terminus of β-NAC and β’-NAC of *S*. *cerevisiae*, and of β-NAC from *C*. *elegans* and *H*. *sapiens*. Amino acids are depicted in the one letter-code. β-NAC of *S*. *cerevisiae* could be aligned completely to the β-sequences of all kingdoms, but the end of the C-terminus of β’ from *S*. *cerevisiae* could not be aligned with the other sequences and was marked as an insert (small letters at the end of the alignment). Colour legend: orange = small hydrophilics, green = small hydrophobics, red = bases, blue = aromatics and colourless = acids/amides and sulphhydrils.

We found major functional differences between αβ-NAC and αβ’-NAC. Only αβ-NAC but not αβ’-NAC (even when expressed at similar levels as αβ-NAC) can suppress all defects found in *nacΔssbΔ* cells including the high sensitivity against translation inhibitory drugs, ribosomal deficiencies that result in halfmer formation, reduced amounts of 80S particles and polysomes, as well as protein aggregation. This suggests that αβ-NAC is functionally most important for yeast vitality. Our results are in agreement with a recent study by Frydman and colleagues analyzing the nascent interactome of NAC [12]. They showed that αβ-NAC has a preference for ribosomes translating metabolic enzymes as well as secretory and membrane proteins while αβ’-NAC preferentially binds to ribosomes translating mitochondrial or ribosomal proteins. This finding implies different substrate pools of αβ-NAC and αβ’-NAC. Both heterodimers are ribosome-associated by the conserved ribosome-binding motif found in β-NAC as well as in β’-NAC (Figs 1B and 7A). In addition, both subunits possess the conserved NAC domain involved in dimerization (Fig 7B). The two different β-subunits display an overall similarity of 64.3% with an identity of 46.5% on their amino acid level. However, β-NAC and β’-NAC obviously reveal strong differences at their C-terminal ends (Fig 7C) as the similarity of this region is only 30.8% with an identity of 10.3%. Both C-termini are predicted to be rather unstructured, however, the C-terminus of β’-NAC is shorter by 8 amino acid residues compared to β-NAC and the last 16 amino acids show no homology to β-NAC at all (Fig 7C). Moreover, β’-NAC has a lower amount of charged amino acids in its C-terminus: 5 negatively charged residues (Asp + Glu) and 2 positively charged residues (Arg + Lys) compared to β-NAC with 11 negatively charged residues (Asp + Glu) and 4 positively charged residues (Arg + Lys). Thus, we speculate that the diverse C-termini of β- and β’-NAC might be involved in substrate selectivity and thus contribute to the functional differences of αβ-NAC and αβ’-NAC. Interestingly, the C-termini of β-NAC subunits from *C*. *elegans* and humans also contain a high number of charged residues and are clearly more similar to yeast β-NAC than to β’-NAC (Fig 7C).

Obviously, αβ-NAC can only display its function *in vivo* when bound to ribosomes and expressed at high levels. This conclusion is strongly supported by the findings that neither αβ^RRK/AAA^-NAC, that forms a stable heterodimer but is deficient in ribosome binding, nor the low expression of αβ-NAC driven by the *BTT1* promoter could complement all phenotypic defects of *nacΔssbΔ* cells.

Interestingly, expression of ribosome-bound β-NAC but not of β’-NAC was sufficient to restore growth but both versions could lower the aggregation propensity of newly synthesized proteins. This suggests some residual chaperoning activities of ribosome-bound β-NAC and β’-NAC subunits, probably as a homodimer which, in the case of β-NAC, is sufficient to promote growth of *nacΔssbΔ* cells.

The α-NAC subunit contains a conserved UBA (ubiquitin–associated) domain at its C-terminal end (Fig 1). UBA domains can bind ubiquitin and thus are often found in proteins associated with the ubiquitin-mediated degradation pathway. However, UBA domains can also act as protein-protein interaction or dimerization surfaces by exposing a hydrophobic patch [31]. That far, no ubiquitin binding of the NAC UBA domain could be demonstrated and the role of this domain is enigmatic. In this study, we discovered a potential role for the NAC UBA domain by studying the α^ΔUBA^β-NAC version, a heterodimer with a wild type (wt) β-NAC subunit and a mutant α-NAC lacking the C-terminal 64 amino acid residues including the complete UBA domain and part of the flexible linker between the UBA and the NAC domain (Fig 1B and 1C). The α^ΔUBA^β-NAC mutant complemented growth defects of *nacΔssbΔ* cells similar to αβ-NAC, but was significantly more potent to suppress protein aggregation in these cells suggesting a higher chaperone activity of α^ΔUBA^β-NAC compared to αβ-NAC. This led us to conclude that the UBA domain attached via a highly flexible linker to the α-NAC subunit may regulate the chaperone activity of αβ-NAC. How can we envision such a role for the UBA domain? We speculate that the hydrophobic and surface-exposed stretch of the UBA domain may contact and transiently cover a hydrophobic substrate binding site in the αβ-NAC heterodimer. Enhanced exposure of such a binding site in α^ΔUBA^β-NAC may allow NAC to associate more efficiently with misfolded proteins to prevent their aggregation. However, such a "hyper" chaperone activity might be harmful as well, e.g. by binding too tight or in an unregulated manner to certain substrates. This may explain why the α^ΔUBA^β-NAC is not as efficient as wt αβ-NAC in suppressing defects in translation. Another possibility would be that the UBA domain contributes to dimerization or oligomerization of αβ-NAC and thereby affects its chaperone activities.

Future *in vivo* and *in vitro* experiments are required to evaluate this interesting hypothesis and to shed more light on the chaperone activity of NAC. This study provides the framework to pursue such analyses.

## Materials and Methods

### Strains, plasmids and growth conditions

The genotypes of the yeast strains are listed in S2 Table. Strains carrying gene deletions were constructed by PCR-based gene disruption [32, 33] and clones were analyzed by PCR. Gene deletions in yeast strains obtained from EUROSCARF were confirmed by growth on YPD-G418 and PCR. All plasmids were generated using standard molecular cloning techniques and are listed in S3 Table. Plasmids encoding wild type (wt) NAC and mutants of NAC were described in [18]. To generate plasmids where the two β-NAC subunits were expressed with the promoter and terminator region originally belonging to the other subunit, the PCR-amplified promoter and terminator regions as well as the coding regions of *EGD1* and *BTT1* (derived from yeast genomic DNA of BY4741) were cloned into pRS316 or pRS316-α-NAC in the desired combinations. All plasmids used are listed in S3 Table. Unless indicated otherwise, yeast cells were grown at 30°C in YPD (1% yeast extract, 2% peptone and 2% dextrose) or defined synthetic complete media (6.7 g/liter YNB, 2 g/liter drop out mix and 2% dextrose). Analysis of cell growth was performed four times.

### Density gradient ultracentrifugation and ribosomal profiling

For yeast ribosome profiling 200 ml cultures in–Ura medium were grown at 30°C to an OD_600_ = 1. Cycloheximide was added to a final concentration of 100 μg/ml. Immediately afterwards, the treated cultures were transferred into centrifuge bottles containing 100 g crushed ice and centrifuged at 5000 x g for 5 min. The cell pellets were frozen in liquid nitrogen and stored at -80°C. Cell pellets were resuspended in lysis buffer (20 mM Hepes-KOH, pH 7.4, 100 mM KAc, 2 mM MgAc, 100 μg/ml cycloheximide, 0.5 mM DTT, 1 mM PMSF, and protease inhibitor cocktail) and 1 g of acid-washed glass beads was added. The cells were lysed mechanically by glass bead disruption. Triton X-100 and sodium deoxycholate were added to a final concentration of 0.25% each after cell lysis. 10 A_260_ absorption units of each lysate in 500 μl volume were loaded onto an 11 ml linear sucrose gradient (15–45% in lysis buffer) and centrifuged for 2 hours at 4°C at 200.000 x g. The gradients were fractionated from top to bottom with a density gradient fractionator (Teledyne Isco, Inc.) and ribosome profiles were monitored at 254 nm. Data were recorded and processed with PeakTrak V1.1 (Teledyne ISco, Inc.). Experiments were performed four times.

### Antibodies and Western Blot analysis

Rabbit polyclonal antibody against the NAC complex was described in [18]; antibodies recognizing PGK1 were obtained from Invitrogen. Protein samples were separated by SDS-PAGE and transferred to a nitrocellulose membrane (GE Healthcare) by Western blotting (wet blot). Primary antibodies were diluted 1:10.000. Fluorescence-labeled secondary anti-rabbit antibodies (DY-682; Dyomics) were applied and visualized with the FLA-9000 system (Fujifilm). Experiments were repeated at least three times.

### RNA isolation and quantitative real-time PCR

Total RNA was isolated from yeast wild type strain using RNeasy Mini Kit (Qiagen) according to the manufacturer’s protocol and transcribed into cDNA according to the QuantiTect reverse transcription kit protocol (Qiagen). For quantitative real-time PCR 20 μl triplicate reactions with 1 μl of 1:5 diluted cDNA were used together with 0.2 mM of the primer pair and 1x GoTaq®qPCR Master Mix (Promega). For detection the ABI 7500 Fast Real-Time PCR System (Applied Biosystems) was used. Data were analyzed using the comparative 2ΔΔCT method and *TAF10* as a reference gene. Experiments were repeated at least three times.

### Isolation of aggregated proteins

Overnight cultures of yeast cells transformed with the indicated plasmids encoding the different NAC subunits were grown in synthetic complete medium without uracil. The main culture was grown in YPD to the logarithmic stage and 50 OD_600_ units were harvested. The cell pellets were resuspended in lysis buffer (20 mM potassium phosphate, pH 6.8, 10 mM DTT, 1 mM EDTA, 0.1% Tween, 1 mM PMSF, protease inhibitor cocktail, 3 mg/ml zymolyase 20T and 25 u/μl DNaseI) and incubated at room temperature for 15 minutes. After chilling on ice for 5 minutes, the samples were treated by tip sonication (Branson, eight times at level 4 and duty cycle 50%) and centrifuged at 4°C for 20 minutes and 200 x g. Protein levels were adjusted to identical concentrations and aggregated proteins were pelleted at 16.000 x g for 20 min at 4°C. Aggregated proteins were washed twice with 2% NP-40 (in 20 mM potassium phosphate, pH 6.8, 1 mM PMSF and protease inhibitor cocktail), sonicated six times at level 4 and 50% duty cycle and centrifuged as described above. The final washing step was performed in buffer without NP-40, samples were sonicated for four times at level 2 with 65% duty cycle and subsequently boiled in SDS sample buffer. The total and aggregated proteins were separated by SDS-PAGE and analyzed by Coomassie staining. Experiments were performed three times. Quantification was performed using ImageJ; mean +/- SD is shown.

### Statistical analysis

Statistical analysis was performed using one-way-between-groups ANOVA with post-hoc Tukey test [29]. All the data from the aggregation experiment were normalized to the mean raw value of the wt data set. A Levenes test for homogeneity of Variance was performed followed by one-Way ANOVA. Tukeys post-hoc test compared the aggregation level of every strain to the other to check for statistically significant differences.

## Supporting Information

S1 Fig mRNA levels of β-NAC subunits are changed when expressed under different promoters. a) Yeast *nacΔssbΔ* mutant cells transformed with the indicated plasmids were grown to an optical density (OD_600_) of 0.8 and mRNA was isolated. cDNA was obtained by reverse transcription and used for qRT-PCR with *EGD1*-specific primer pairs. The samples were normalized to an internal control (housekeeping gene) and compared to wild type. b) Experiment performed as in a) with *BTT1*-specific primer pairs.
(TIF)

S2 Fig Equalized protein levels of lysates used for aggregate isolation. a) 50 OD_600_ units of transformed yeast cells in the logarithmic phase were lysed and the aggregated protein material was quantitatively isolated. 15 μg of total lysates were separated by SDS-PAGE and visualized by Coomassie staining. b) Total lysates prepared in a) were used for Western blotting to analyse the expression levels of the different NAC-encoding plasmids. Pgk1 served as loading control. The asterisks mark a degradation product of α-NAC (*) and an unspecific protein band (**).
(TIF)

S1 Table The levels of aggregated proteins were analysed by one-way-between-groups ANOVA followed by Tukeys test. ANOVA Statistics: One-way-between-groups ANOVA assessed a statistically significant divergence of the portions of aggregated proteins between the different strains. Tukey honest significant difference comparison: the levels of isolated aggregates of each strain were compared with the ones of the respective other strain to test for statistically significant differences.
(DOCX)

S2 Table Yeast strains used in this study. All yeast strains used in this work were isogenic derivatives of BY4741 and are listed by their genotypes.
(DOCX)

S3 Table Plasmids used for complementation studies of the *nacΔssbΔ* phenotypes. All plasmids were generated using standard molecular cloning techniques.
(DOCX)

## References

[1] Bukau B, Weissman J, Horwich A. Molecular chaperones and protein quality control. Cell. 2006;125: 443–451. 10.1016/j.cell.2006.04.014 . 16678092

[2] Preissler S, Deuerling E. Ribosome-associated chaperones as key players in proteostasis. Trends Biochem Sci. 2012;37: 274–283. 10.1016/j.tibs.2012.03.002 . 22503700

[3] Wang S, Sakai H, Wiedmann M. NAC covers ribosome-associated nascent chains thereby forming a protective environment for regions of nascent chains just emerging from the peptidyl transferase center. J Cell Biol. 1995;130: 519–528. 7622554 10.1083/jcb.130.3.519 PMC2120527

[4] Spreter T, Pech M, Beatrix B. The crystal structure of archaeal nascent polypeptide-associated complex (NAC) reveals a unique fold and the presence of a ubiquitin-associated domain. J Biol Chem. 2005;280: 15849–15854. 10.1074/jbc.M500160200 . 15665334

[5] Wang L, Zhang W, Wang L, Zhang XC, Li X, Rao Z. Crystal structures of NAC domains of human nascent polypeptide-associated complex (NAC) and its alphaNAC subunit. Protein Cell. 2010;1: 406–416. 10.1007/s13238-010-0049-3 . 21203952 PMC4875098

[6] Liu Y, Hu Y, Li X, Niu L, Teng M. The crystal structure of the human nascent polypeptide-associated complex domain reveals a nucleic acid-binding region on the NACA subunit. Biochemistry. 2010;49: 2890–2896. 10.1021/bi902050p . 20214399

[7] Wiedmann B, Sakai H, Davis TA, Wiedmann M. A protein complex required for signal-sequence-specific sorting and translocation. Nature. 1994;370: 434–440. 10.1038/370434a0 . 8047162

[8] Franke J, Reimann B, Hartmann E, Kohlerl M, Wiedmann B. Evidence for a nuclear passage of nascent polypeptide-associated complex subunits in yeast. J Cell Sci. 2001;114: 2641–2648. . 11683391 10.1242/jcs.114.14.2641

[9] Wegrzyn RD, Hofmann D, Merz F, Nikolay R, Rauch T, Graf C, et al A conserved motif is prerequisite for the interaction of NAC with ribosomal protein L23 and nascent chains. J Biol Chem. 2006;281: 2847–2857. 10.1074/jbc.M511420200 . 16316984

[10] Nyathi Y, Pool MR. Analysis of the interplay of protein biogenesis factors at the ribosome exit site reveals new role for NAC. J Cell Biol. 2015;210: 287–301. 10.1083/jcb.201410086 26195668 PMC4508901

[11] Pech M, Spreter T, Beckmann R, Beatrix B. Dual binding mode of the nascent polypeptide-associated complex reveals a novel universal adapter site on the ribosome. J Biol Chem. 2010;285: 19679–19687. Epub 2010/04/23. 10.1074/jbc.M109.092536 20410297 PMC2885246

[12] del Alamo M, Hogan DJ, Pechmann S, Albanese V, Brown PO, Frydman J. Defining the specificity of cotranslationally acting chaperones by systematic analysis of mRNAs associated with ribosome-nascent chain complexes. PLoS Biol. 2011;9: e1001100 10.1371/journal.pbio.1001100 21765803 PMC3134442

[13] George R, Beddoe T, Landl K, Lithgow T. The yeast nascent polypeptide-associated complex initiates protein targeting to mitochondria in vivo. Proc Natl Acad Sci U S A. 1998;95: 2296–2301. 9482879 10.1073/pnas.95.5.2296 PMC19325

[14] Reimann B, Bradsher J, Franke J, Hartmann E, Wiedmann M, Prehn S, et al Initial characterization of the nascent polypeptide-associated complex in yeast. Yeast. 1999;15: 397–407. 10.1002/(SICI)1097-0061(19990330)15:5<397::AID-YEA384>3.0.CO;2-U . 10219998

[15] Bloss TA, Witze ES, Rothman JH. Suppression of CED-3-independent apoptosis by mitochondrial betaNAC in Caenorhabditis elegans. Nature. 2003;424: 1066–1071. 10.1038/nature01920 . 12944970

[16] Deng JM, Behringer RR. An insertional mutation in the BTF3 transcription factor gene leads to an early postimplantation lethality in mice. Transgenic Res. 1995;4: 264–269. . 7655515 10.1007/BF01969120

[17] Markesich DC, Gajewski KM, Nazimiec ME, Beckingham K. Bicaudal encodes the *Drosophila* beta NAC homolog, a component of the ribosomal translational machinery. Development. 2000;127: 559–572. . 10631177 10.1242/dev.127.3.559

[18] Koplin A, Preissler S, Ilina Y, Koch M, Scior A, Erhardt M, et al A dual function for chaperones SSB-RAC and the NAC nascent polypeptide-associated complex on ribosomes. J Cell Biol. 2010;189: 57–68. 10.1083/jcb.200910074 20368618 PMC2854369

[19] Kirstein-Miles J, Scior A, Deuerling E, Morimoto RI. The nascent polypeptide-associated complex is a key regulator of proteostasis. EMBO J. 2013;32: 1451–1468. 10.1038/emboj.2013.87 23604074 PMC3655472

[20] Gamerdinger M, Hanebuth MA, Frickey T, Deuerling E. The principle of antagonism ensures protein targeting specificity at the endoplasmic reticulum. Science. 2015;348: 201–207. 10.1126/science.aaa5335 . 25859040

[21] Wiedmann B, Prehn S. The nascent polypeptide-associated complex (NAC) of yeast functions in the targeting process of ribosomes to the ER membrane. FEBS Lett. 1999;458: 51–54. . 10518932 10.1016/s0014-5793(99)01118-7

[22] Sydorskyy Y, Dilworth DJ, Yi EC, Goodlett DR, Wozniak RW, Aitchison JD. Intersection of the Kap123p-mediated nuclear import and ribosome export pathways. Mol Cell Biol. 2003;23: 2042–2054. 12612077 10.1128/MCB.23.6.2042-2054.2003 PMC149464

[23] Basu U, Si K, Warner JR, Maitra U. The Saccharomyces cerevisiae TIF6 gene encoding translation initiation factor 6 is required for 60S ribosomal subunit biogenesis. Mol Cell Biol. 2001;21: 1453–1462. 10.1128/MCB.21.5.1453-1462.2001 11238882 PMC86691

[24] Gorenstein C, Warner JR. Coordinate regulation of the synthesis of eukaryotic ribosomal proteins. Proc Natl Acad Sci U S A. 1976;73: 1547–1551. 775493 10.1073/pnas.73.5.1547 PMC430334

[25] Lopez N, Halladay J, Walter W, Craig EA. SSB, encoding a ribosome-associated chaperone, is coordinately regulated with ribosomal protein genes. J Bacteriol. 1999;181: 3136–3143. 10322015 10.1128/jb.181.10.3136-3143.1999 PMC93769

[26] Albanese V, Yam AY, Baughman J, Parnot C, Frydman J. Systems analyses reveal two chaperone networks with distinct functions in eukaryotic cells. Cell. 2006;124: 75–88. 10.1016/j.cell.2005.11.039 . 16413483

[27] Meyer AE, Hung NJ, Yang P, Johnson AW, Craig EA. The specialized cytosolic J-protein, Jjj1, functions in 60S ribosomal subunit biogenesis. Proc Natl Acad Sci U S A. 2007;104: 1558–1563. 10.1073/pnas.0610704104 17242366 PMC1785244

[28] Demoinet E, Jacquier A, Lutfalla G, Fromont-Racine M. The Hsp40 chaperone Jjj1 is required for the nucleo-cytoplasmic recycling of preribosomal factors in Saccharomyces cerevisiae. RNA. 2007;13: 1570–1581. 10.1261/rna.585007 17652132 PMC1950757

[29] Wessa P. Free Statistics Software, Office for Research Development and Education [URL]. 2015. Available: http://www.wessa.net/.

[30] Rospert S, Dubaquie Y, Gautschi M. Nascent-polypeptide-associated complex. Cell Mol Life Sci. 2002;59: 1632–1639. . 12475173 10.1007/PL00012490 PMC11337418

[31] Hartmann-Petersen R, Semple CA, Ponting CP, Hendil KB, Gordon C. UBA domain containing proteins in fission yeast. Int J Biochem Cell Biol. 2003;35: 629–636. . 12672455 10.1016/s1357-2725(02)00393-x

[32] Brachmann CB, Davies A, Cost GJ, Caputo E, Li J, Hieter P, et al Designer deletion strains derived from Saccharomyces cerevisiae S288C: a useful set of strains and plasmids for PCR-mediated gene disruption and other applications. Yeast. 1998;14: 115–132. 10.1002/(SICI)1097-0061(19980130)14:2<115::AID-YEA204>3.0.CO;2-2 . 9483801

[33] Goldstein AL, McCusker JH. Three new dominant drug resistance cassettes for gene disruption in Saccharomyces cerevisiae. Yeast. 1999;15: 1541–1553. Epub 1999/10/09. 10.1002/(SICI)1097-0061(199910)15:14<1541::AID-YEA476>3.0.CO;2-K . 10514571

[34] Frickey T, Lupas A. CLANS: a Java application for visualizing protein families based on pairwise similarity. Bioinformatics. 2004;20: 3702–3704. 10.1093/bioinformatics/bth444 . 15284097

[35] Edgar RC. MUSCLE: multiple sequence alignment with high accuracy and high throughput. Nucleic Acids Res. 2004;32: 1792–1797. 10.1093/nar/gkh340 15034147 PMC390337

