# Supplementary figures and images for: Functional Dissection of the Nascent Polypeptide-Associated Complex in Saccharomyces cerevisiae
Source: PLoS One. 2015 Nov 30;10(11):e0143457. doi: 10.1371/journal.pone.0143457 (PMC4664479; doi:10.1371/journal.pone.0143457)

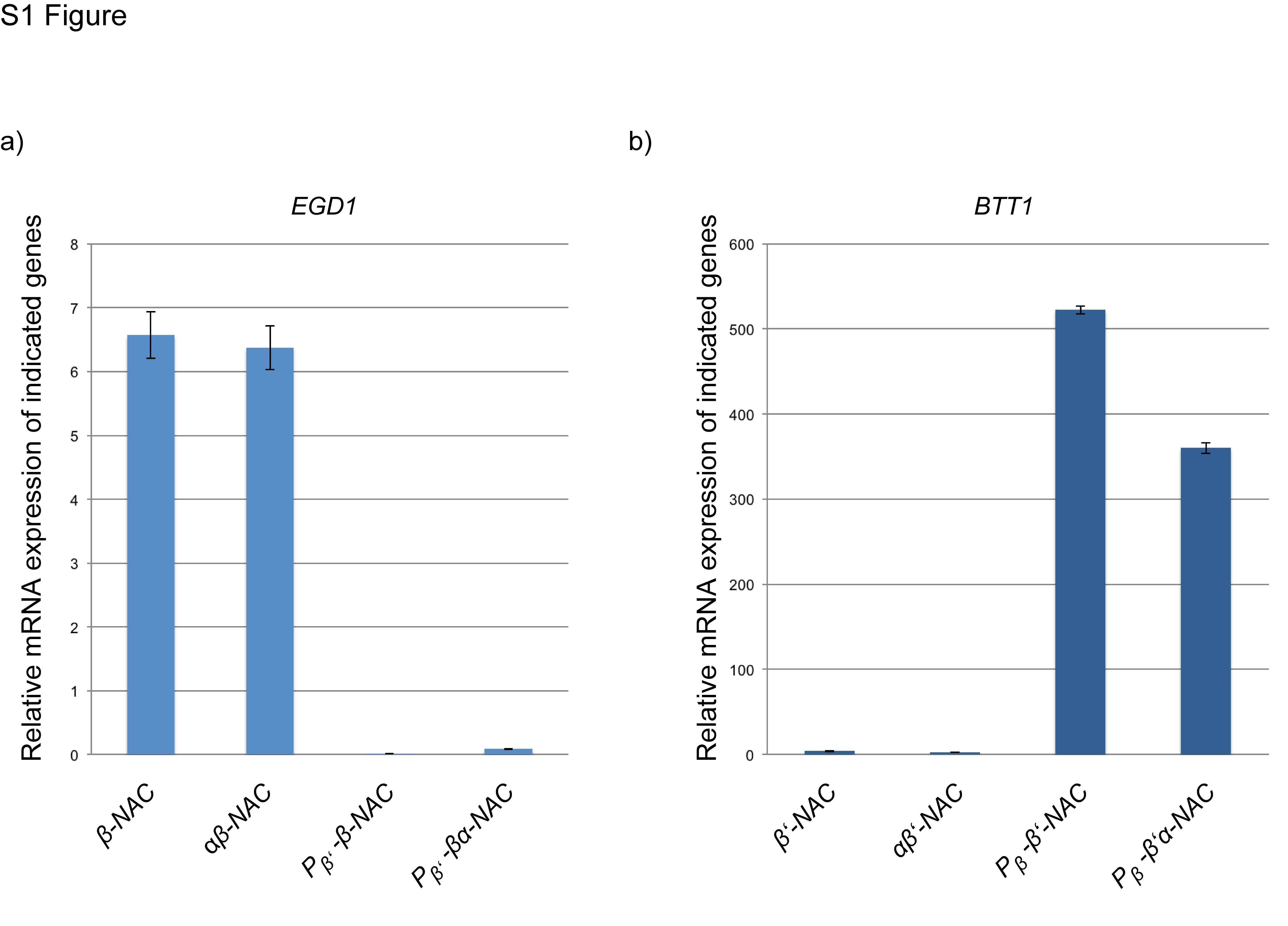

Supplement: S1 Fig — a) Yeast nacΔssbΔ mutant cells transformed with the indicated plasmids were grown to an optical density (OD600) of 0.8 and mRNA was isolated. cDNA was obtained by reverse transcription and used for qRT-PCR with EGD1-specific primer pairs. The samples were normalized to an internal control (housekeeping gene) and compared to wild type. b) Experiment performed as in a) with BTT1-specific primer pairs. (TIF) [file pone.0143457.s001.tif]

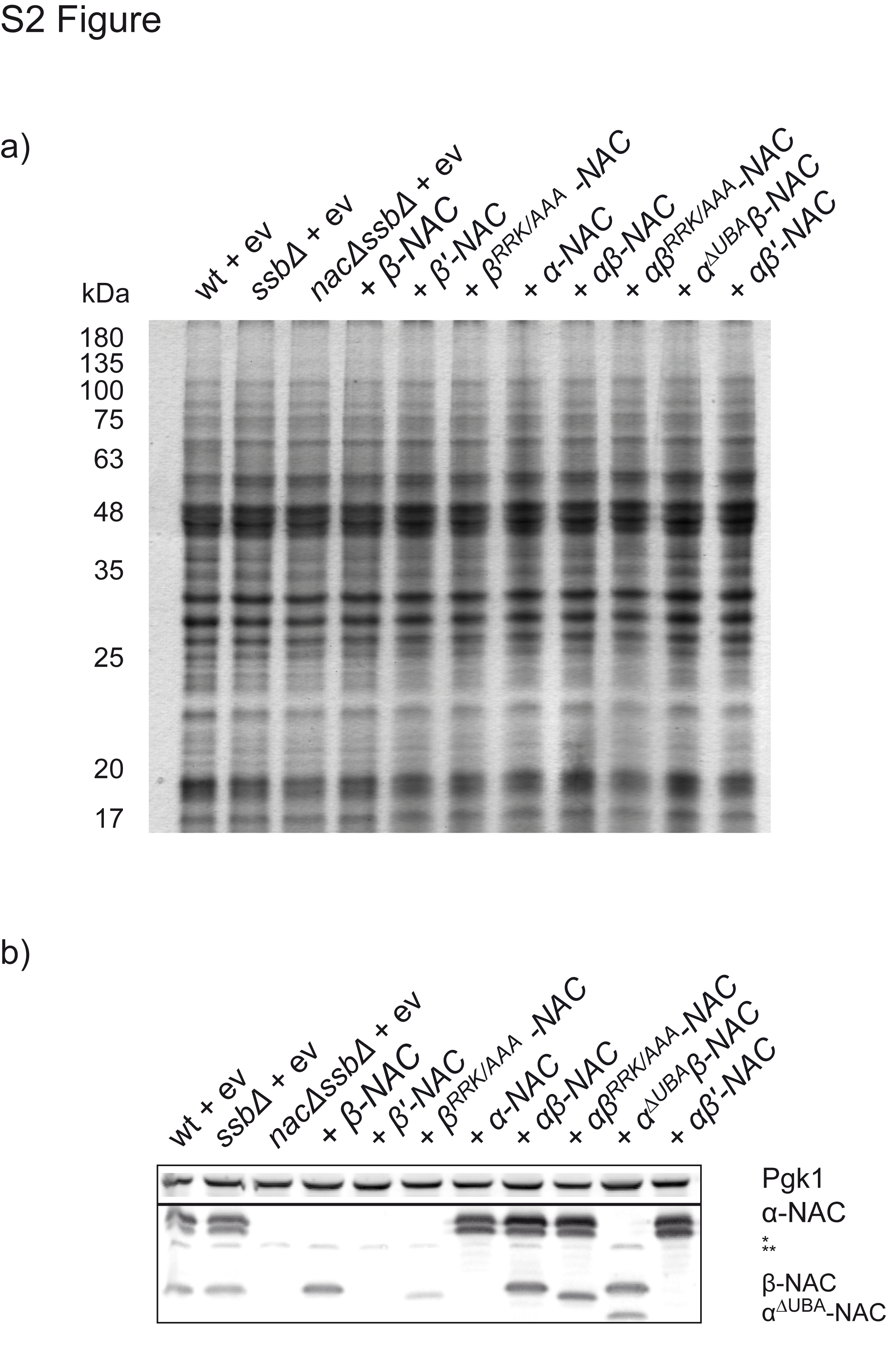

Supplement: S2 Fig — a) 50 OD600 units of transformed yeast cells in the logarithmic phase were lysed and the aggregated protein material was quantitatively isolated. 15 μg of total lysates were separated by SDS-PAGE and visualized by Coomassie staining. b) Total lysates prepared in a) were used for Western blotting to analyse the expression levels of the different NAC-encoding plasmids. Pgk1 served as loading control. The asterisks mark a degradation product of α-NAC (*) and an unspecific protein band (**). (TIF) [file pone.0143457.s002.tif]
