# Supplementary material for: Functional Dissection of the Nascent Polypeptide-Associated Complex in Saccharomyces cerevisiae
Source: PLoS One. 2015 Nov 30;10(11):e0143457. doi: 10.1371/journal.pone.0143457 (PMC4664479; doi:10.1371/journal.pone.0143457)
Supplement: S1 Table — ANOVA Statistics: One-way-between-groups ANOVA assessed a statistically significant divergence of the portions of aggregated proteins between the different strains. Tukey honest significant difference comparison: the levels of isolated aggregates of each strain were compared with the ones of the respective other strain to test for statistically significant differences. (DOCX) [file pone.0143457.s003.docx]

**S1 Table: The levels of aggregated proteins were analyzed by one-way-between-groups ANOVA followed by Tukeys test.**

| **Levenes Test For Homogeneity of Variance** | |
| --- | --- |
| F-value | Pr (>F) |
| 0.587 | 0.807 |

| **ANOVA Statistics** | |
| --- | --- |
| F-value | Pr (>F) |
| 13.932 | 0 |

| **Tukey Honest Significant Difference Comparison** | |
| --- | --- |
| Strain | p-value |
| *ssb∆* to wild type | 0.015 |
| *nac∆ssb∆* to wild type | 0 |
| *nac∆ssb∆* + *β-NAC* to wild type | 0.007 |
| *nac∆ssb∆* + *β'-NAC* to wild type | 0 |
| *nac∆ssb∆* + *β^RRK/AAA^-NAC* to wild type | 0 |
| *nac∆ssb∆* + *α-NAC* to wild type | 0 |
| *nac∆ssb∆* + *αβ-NAC* to wild type | 0.042 |
| *nac∆ssb∆* + *αβ^RRK/AAA^-NAC* to wild type | 0 |
| *nac∆ssb∆* + *α^∆UBA^*β*-NAC* to wild type | 0.633 |
| *nac∆ssb∆* + *αβ'-NAC* to wild type | 0.001 |
| *nac∆ssb∆* to *ssb∆* | 0.001 |
| *nac∆ssb∆* + *β-NAC* to *ssb∆* | 1 |
| *nac∆ssb∆* + *β'-NAC* to *ssb∆* | 0.805 |
| *nac∆ssb∆* + *β^RRK/AAA^-NAC* to *ssb∆* | 0.027 |
| *nac∆ssb∆* + *α-NAC* to *ssb∆* | 0.844 |
| *nac∆ssb∆* + *αβ-NAC* to *ssb∆* | 1 |
| *nac∆ssb∆* + *αβ^RRK/AAA^-NAC* to *ssb∆* | 0.327 |
| *nac∆ssb∆* + *α^∆UBA^*β*-NAC* to *ssb∆* | 0.584 |
| *nac∆ssb∆* + *αβ'-NAC* to *ssb∆* | 0.992 |
| *nac∆ssb∆* + *β-NAC* to *nac∆ssb∆* | 0.002 |
| *nac∆ssb∆* + *β'-NAC* to *nac∆ssb∆* | 0.05 |
| *nac∆ssb∆* + *β^RRK/AAA^-NAC* to *nac∆ssb∆* | 0.921 |
| *nac∆ssb∆* + *α-NAC* to *nac∆ssb∆* | 0.042 |
| *nac∆ssb∆* + *αβ-NAC* to *nac∆ssb∆* | 0 |
| *nac∆ssb∆* + *αβ^RRK/AAA^-NAC* to *nac∆ssb∆* | 0.24 |
| *nac∆ssb∆* + *α^∆UBA^*β*-NAC* to *nac∆ssb∆* | 0 |
| *nac∆ssb∆* + *αβ'-NAC* to *nac∆ssb∆* | 0.011 |
| *nac∆ssb∆* + *β'-NAC* to *nac∆ssb∆ + β-NAC* | 0.93 |
| *nac∆ssb∆* + *β^RRK/AAA^-NAC* to *nac∆ssb∆ + β-NAC* | 0.053 |
| *nac∆ssb∆* + *α-NAC* to *nac∆ssb∆ + β-NAC* | 0.95 |
| *nac∆ssb∆* + *αβ-NAC* to *nac∆ssb∆ + β-NAC* | 0.999 |
| *nac∆ssb∆* + *αβ^RRK/AAA^-NAC* to *nac∆ssb∆ + β-NAC* | 0.499 |
| *nac∆ssb∆* + *α^∆UBA^β-NAC* to *nac∆ssb∆ + β-NAC* | 0.399 |
| *nac∆ssb∆* + *αβ'-NAC* to *nac∆ssb∆ + β-NAC* | 1 |
| *nac∆ssb∆* + *β^RRK/AAA^-NAC* to *nac∆ssb∆ + β’-NAC* | 0.572 |
| *nac∆ssb∆* + *α-NAC* to *nac∆ssb∆ + β’-NAC* | 1 |
| *nac∆ssb∆* + *αβ-NAC* to *nac∆ssb∆ + β’-NAC* | 0.52 |
| *nac∆ssb∆* + *αβ^RRK/AAA^-NAC* to *nac∆ssb∆ + β’-NAC* | 0.999 |
| *nac∆ssb∆* + *α^∆UBA^β-NAC* to *nac∆ssb∆* + *β’-NAC* | 0.028 |
| *nac∆ssb∆* + *αβ'-NAC* to *nac∆ssb∆ + β’-NAC* | 1 |
| *nac∆ssb∆* + *α-NAC* to *nac∆ssb∆* + *β^RRK/AAA^-NAC* | 0.523 |
| *nac∆ssb∆* + *αβ-NAC* to *nac∆ssb∆* + *β^RRK/AAA^-NAC* | 0.009 |
| *nac∆ssb∆* + *αβ^RRK/AAA^-NAC* to *nac∆ssb∆* + *β^RRK/AAA^-NAC* | 0.959 |
| *nac∆ssb∆* + *α^∆UBA^*β*-NAC* to *nac∆ssb∆* + *β^RRK/AAA^-NAC* | 0 |
| *nac∆ssb∆* + *αβ'-NAC* to *nac∆ssb∆* + *β^RRK/AAA^-NAC* | 0.212 |
| *nac∆ssb∆* + *αβ-NAC* to *nac∆ssb∆* + *α-NAC* | 0.57 |
| *nac∆ssb∆* + *αβ^RRK/AAA^-NAC* to *nac∆ssb∆* + *α-NAC* | 0.997 |
| *nac∆ssb∆* + *α^∆UBA^*β*-NAC* to *nac∆ssb∆* + *α-NAC* | 0.034 |
| *nac∆ssb∆* + *αβ'-NAC* to *nac∆ssb∆* + *α-NAC* | 1 |
| *nac∆ssb∆* + *αβ^RRK/AAA^-NAC* to *nac∆ssb∆* + *αβ-NAC* | 0.145 |
| *nac∆ssb∆* + *α^∆UBA^*β*-NAC* to *nac∆ssb∆* + *αβ-NAC* | 0.854 |
| *nac∆ssb∆* + *αβ'-NAC* to *nac∆ssb∆* + *αβ-NAC* | 0.9 |
| *nac∆ssb∆* + *α^∆UBA^*β*-NAC* to *nac∆ssb∆* + *αβ^RRK/AAA^* | 0.004 |
| *nac∆ssb∆* + *αβ'-NAC* to *nac∆ssb∆* + *αβ^RRK/AAA^ -NAC* | 0.897 |
| *nac∆ssb∆* + αβ*'-NAC* to *ssb∆* to *nac∆ssb∆* + *α^∆UBA^*β*-NAC* | 0.119 |
