# Supplementary material for: Functional Dissection of the Nascent Polypeptide-Associated Complex in Saccharomyces cerevisiae
Source: PLoS One. 2015 Nov 30;10(11):e0143457. doi: 10.1371/journal.pone.0143457 (PMC4664479; doi:10.1371/journal.pone.0143457)
Supplement: S2 Table — All yeast strains used in this work were isogenic derivatives of BY4741 and are listed by their genotypes. (DOCX) [file pone.0143457.s004.docx]

| **Yeast strain** | **Genotype** | **Reference** |
| --- | --- | --- |
| BY4741 | *MAT***a**; *his3Δ1*; *leu2Δ0*; *met15Δ0*; *ura3Δ0* | EUROSCARF |
| *ssbΔ* | *MAT***a**; *his3Δ1*; *leu2Δ0*; *met15Δ0*; *ura3Δ0*; *ssb1::kanMX4*; *ssb2::natMX4* | (Koplin et al, 2010) |
| *nacΔssbΔ* | *MAT***a**; *his3Δ1*; *leu2Δ0*; *met15Δ0*; *ura3Δ0*; *ssb1::kanMX4*; *ssb2::natMX4; egd1::hisMX6; btt1::phleo; egd2::LEU2* | (Koplin et al, 2010) |
| *jjj1Δ* | *MAT***a**; *his3Δ1*; *leu2Δ0*; *met15Δ0*; *ura3Δ0; jjj1::kanMX4* | EUROSCARF |
| *nacΔjjj1Δ* | *MAT***a**; *his3Δ1*; *leu2Δ0*; *met15Δ0*; *ura3Δ0; egd1::hisMX6; btt1::phleo; egd2::LEU2* | This study |

**S2 Table: Yeast strains used in this study.**
