# Supplementary material for: Functional Dissection of the Nascent Polypeptide-Associated Complex in Saccharomyces cerevisiae
Source: PLoS One. 2015 Nov 30;10(11):e0143457. doi: 10.1371/journal.pone.0143457 (PMC4664479; doi:10.1371/journal.pone.0143457)
Supplement: S3 Table — All plasmids were generated using standard molecular cloning techniques. (DOCX) [file pone.0143457.s005.docx]

**S3 Table: Plasmids used for complementation studies of the *nac∆ssb∆* phenotypes.**

| **Plasmid** | **Inserted gene/ORF** | **Promoter** | **Basic vector** | **Reference** |
| --- | --- | --- | --- | --- |
| pRS316 | None | None | pRS316 | Sikorsky & Hieter, 1989 |
| β-NAC | *EGD1* | *EGD1* | pRS316 | Koplin et al., 2010 |
| β'-NAC | *BTT1* | *BTT1* | pRS316 | This study |
| β^RRK/AAA^-NAC | *EGD1* | *EGD1* | pRS316 | Koplin et al., 2010 |
| αβ-NAC | *EGD1, EGD2* | *EGD1, EGD2* | pRS316 | Koplin et al., 2010 |
| αβ^RRK/AAA^-NAC | *EGD1, EGD2* | *EGD1, EGD2* | pRS316 | Koplin et al., 2010 |
| α^∆UBA^β-NAC | *EGD1, EGD2* | *EGD1, EGD2* | pRS316 | This study |
| P_β’_-β-NAC | *EGD1* | *BTT1* | pRS316 | This study |
| P_β_-β’-NAC | *BTT1* | *EGD1* | pRS316 | This study |
| αβ'-NAC | *BTT1, EGD2* | *BTT1, EGD2* | pRS316 | This study |
| P_β’_-αβ-NAC | *EGD1, EGD2* | *BTT1, EGD2* | pRS316 | This study |
| P_β_-αβ’-NAC | *BTT1, EGD2* | *EGD1, EGD2* | pRS316 | This study |
